# Supplementary material for: Pregnant women’s unmet need to communicate with a health professional during the SARS-CoV-2 pandemic lockdown in France: The Covimater cross-sectional study
Source: PLoS One. 2022 Apr 28;17(4):e0266996. doi: 10.1371/journal.pone.0266996 (PMC9049552; doi:10.1371/journal.pone.0266996)
Supplement: Attachment — Submitted filename: Response to Reviewers_vf.docx [file pone.0266996.s001.docx]

**Authors’ response to the Reviewers’ comments**

Plos One

Title : Pregnant women's unmet need to communicate with a health professional during the SARS-CoV-2 pandemic in France: the Covimater cross-sectional study
PONE-D-21-31849

**Reviewer #1:**

**General response to reviewer #1** : The questions posed by reviewer #1 and in particular the proposed bibliography (sometimes not listed in PubMed) refer to topics related to economics, finance, business, impact of the Covid-19 pandemic on tourism and therefore do not correspond to our subject of analysis. We assume that these remarks correspond to another article. They will therefore not be dealt with here.

I am glad to assess this study entitled, " Pregnant women's unmet need to communicate with a health professional during the SARS-CoV-2 pandemic in France: the Covimater cross-sectional study."
I suggest some minor corrections to check the typo errors in writing to enhance the English quality to reach the scientific merit for the publication of this study. I will this article after the following modifications.

This present study explains that
When the pandemic started, healthcare systems throughout the world rapidly reorganised to respond to what was an unprecedented situation. We aimed to i) describe the unmet need of pregnant women living in France to communicate with health professionals about the pandemic and their pregnancy, ii) assess the socio demographic, medical and contextual factors associated with this unmet need I am in favor of this study and will recommend for publication. However, the authors need to revise the manuscript and work according to my suggestions to enhance the quality. I will accept this paper for publication after these minor changes as suggested below.

Introduction and literation sections

I recommend the authors add suggested articles in the introduction and literature sections. These research articles have identified health-related topics I believe it will improve the quality of your work. I strongly suggested them to improve this section a bit more. I advise authors to revisit their introduction and literature sections of the recommended studies and cite these studies to enhance your research study's quality to reach scientific merit for publication.

Wang, C., Wang, D., Abbas, J., Duan, K., & Mubeen, R. (2021). Global financial crisis, smart lockdown strategies, and the COVID-19 spillover impacts: A global perspective implications from Southeast Asia. Front Psychiatry, 12, 1-14. doi:10.3389/fpsyt.2021.643783

Abbas, J., Hussain, I., Hussain, S., Akram, S., Shaheen, I., & Niu, B. (2019). The Impact of Knowledge Sharing and Innovation upon Sustainable Performance in Islamic Banks: A Mediation Analysis through an SEM Approach. Sustainability, 11(15), 4049. doi:10.3390/su11154049

NeJhaddadgar, N., Ziapour, A., Zakkipour, G., Abolfathi, M., & Shabani, M. (2020, Nov 13). Effectiveness of telephone-based screening and triage during COVID-19 outbreak in the promoted primary healthcare system: a case study in Ardabil province, Iran. Z Gesundh Wiss, 1-6. <https://doi.org/10.1007/s10389-020-01407-8>

Literature
I want to see publish this creative study after some corrections. I have endorsed this study as; it deserves the merit for publication. However, I suggest the authors make minor corrections according to my advice. Please read the suggested studies and cite them in the introduction, literature, and method sections. How corporate social responsibility, innovation and social media and internet use is helpful. Add few lines in the introduction and literature sections. How companies are practicing CSR, business, entrepreneurial networks with innovation and knowledge sharing to improve the business performance and provide better healthcare medicines?

Abbas, J., Raza, S., Nurunnabi, M., Minai, M. S., & Bano, S. (2019). The Impact of Entrepreneurial Business Networks on Firms’ Performance Through a Mediating Role of Dynamic Capabilities. Sustainability, 11(11), 3006. doi:10.3390/su11113006

Azizi, M. R., Atlasi, R., Ziapour, & Naemi, R. (2021). Innovative human resource management strategies during the COVID-19 pandemic: A systematic narrative review approach. Heliyon, 7(6), e07233. doi:10.1016/j.heliyon.2021.e07233

Abbas, J., Zhang, Q., Hussain, I., Akram, S., Afaq, A., & Shad, M. A. (2020). Sustainable Innovation in Small Medium Enterprises: The Impact of Knowledge Management on Organizational Innovation through a Mediation Analysis by Using SEM Approach. Sustainability, 12(6), 2407. doi:https://doi.org/10.3390/su12062407

Azadi, N. A., Ziapour, A., Lebni, J. Y., Irandoost, S. F., & Chaboksavar, F. (2021). The effect of education based on health belief model on promoting preventive behaviors of hypertensive disease in staff of the Iran University of Medical Sciences. Archives of Public Health, 79(1), 69. doi:10.1186/s13690-021-00594-4

Materials and Methods
The results section of the paper presents a good view of the study. This work presents a notable investigation on a selected topic. I suggest the authors to present high quality graphs. By including some graphical presentations will improve the quality of this study. Please see the proposed studies and see the graphical representation. Improve your work like these studies and cite them in this section.

Paulson, K. R., Kamath, A. M., Alam, T., Bienhoff, K., Abady, G. G., . . . Kassebaum, N. J. (2021). Global, regional, and national progress towards Sustainable Development Goal 3.2 for neonatal and child health: all-cause and cause-specific mortality findings from the Global Burden of Disease Study 2019. The Lancet, 1-36. doi:10.1016/s0140-6736(21)01207-1

Hussain, T., Wei, Z., & Nurunnabi, M. (2019). The Effect of Sustainable Urban Planning and Slum Disamenity on The Value of Neighboring Residential Property: Application of The Hedonic Pricing Model in Rent Price Appraisal. Sustainability, 11(4), 1144. doi:10.3390/su11041144

Abbas, J., Aman, J., Nurunnabi, M., & Bano, S. (2019). The Impact of Social Media on Learning Behavior for Sustainable Education: Evidence of Students from Selected Universities in Pakistan. Sustainability, 11(6). <https://doi.org/10.3390/su11061683>

Mubeen, R., Han, D., Abbas, J., & Hussain, I. (2020). The Effects of Market Competition, Capital Structure, and CEO Duality on Firm Performance: A Mediation Analysis by Incorporating the GMM Model Technique. Sustainability, 12(8), 3480. doi:10.3390/su12083480

Discussion
I suggest the authors to discuss the effects of the COVID-19. I suggest you to cite these studies. Read the proposed studies to improve your results and discussion section. See the recommended studies and improve your sections. How companies are practicing CSR, business, entrepreneurial networks with innovation and knowledge sharing to improve the business performance and provide better performance?
Su, Z., McDonnell, D., Wen, J., Kozak, M., Šegalo, S., . . . Xiang, Y.-T. (2021). Mental health consequences of COVID-19 media coverage: the need for effective crisis communication practices. Globalization and Health, 17(1), 4. doi:10.1186/s12992-020-00654-4

Abbas, J., Mahmood, S., Ali, H., Ali Raza, M., Ali, G., Aman, J., . . . Nurunnabi, M. (2019). The Effects of Corporate Social Responsibility Practices and Environmental Factors through a Moderating Role of Social Media Marketing on Sustainable Performance of Firms’ Operating in Multan, Pakistan. Sustainability, 11(12), 3434. doi:10.3390/su11123434

Aqeel, M., Shuja, K. H., Rehna, T., Ziapour, A., Yousaf, I., & Karamat, T. (2021). The Influence of Illness Perception, Anxiety and Depression Disorders on Students Mental Health during COVID-19 Outbreak in Pakistan: A Web-Based Cross-Sectional Survey. International Journal of Human Rights in Healthcare, 14, 1-14.

Abbas, J. (2020). The Impact of Coronavirus (SARS-CoV2) Epidemic on Individuals Mental Health: The Protective Measures of Pakistan in Managing and Sustaining Transmissible Disease. Psychiatr Danub, 32(3-4), 472-477. <https://doi.org/10.24869/psyd.2020.472>

Conclusion
I suggest you make a separate heading of the conclusion and do not mix it with implications.

Policy Recommendations
I again recommend you to make a separate heading of the Policy Recommendations.

The conclusion section is acceptable. Overall, this presents a good piece of research work. I recommend that authors do a little more work and revise this article accordingly. I suggest the authors check English quality and fix some weak sentences. If you have already taken English editing service, ask them to recheck the quality to meet scientific merit for publication. I endorse this manuscript for publication after minor corrections, as suggested.


Abbasi, K. R., Abbas, J., & Tufail, M. (2021). Revisiting electricity consumption, price, and real GDP: A modified sectoral level analysis from Pakistan. Energy Policy, 149, 112087. doi:10.1016/j.enpol.2020.112087

Local Burden of Disease, H. I. V. C. (2021, 2021/01/08). Mapping subnational HIV mortality in six Latin American countries with incomplete vital registration systems. BMC Medicine, 19(1), 4. <https://doi.org/10.1186/s12916-020-01876-4>

Pay attention of English quality to reach scientific merit. I suggest to cite these six studies to improve the quality of the Introduction, Literature and Methods sections. I accept and endorse this manuscript for publication after the suggested minor corrections.

**Reviewer #2:**

**Abstract:**

1) Add a short background explaining the study question.

**Answer 1:**

We modified the abstract as follows:

*o	(page 2-line 26)*: “*During the severe acute respiratory syndrome coronavirus 2 (SARS-CoV-2) pandemic lockdown, communication between pregnant women and health professionals may have become complicated due to restrictions on movement and the saturation of national health systems. This could have impacts on pregnancy monitoring and women’s wellbeing.”*

2) The period of the study is confusing "July 2020" and "March to May 2020". Clarify

**Answer :**

Thank you for this question that underlines the need for clarification. The Covimater study is a cross-sectional study that was carried out in July 2020. It concerned the retrospective experience of 500 pregnant women during the first lockdown in France i.e. March to May 2020. The study period is therefore the first lockdown (March-May 2020). In order to clarify, we modified the abstract as follows:

*o	(page 2-line 38)*: “*The Covimater cross-sectional study conducted in July 2020 includes data from the first lockdown experience of 500 adult women who were pregnant during this period in France (i.e. from March to May 2020)*.”

3) Add detailed data about the participants and their criteria.

**Answer :**
In addition to the fact that the women were adults (>=18 years old) and that they were pregnant during the study period, we specified that the women resided in France.

*o	(page 2-line 41): “The women, all residents in metropolitan France, answered a web-based questionnaire about their conversations with health professionals …”.*

4) The conclusion should be precise. Add the future directions.

**Answer :**
We modified the abstract as follows:

*o	(page 3-line 59): “*These results can be used to develop targeted strategies that ensure pregnant women are able to i) communicate with health professionals*…”*

**Introduction:**

1) Define "SARS-COV-2 and its negative effects on pregnant women" in detail.

**Answer 1:**
We modified the manuscript as follows in order to define SARS-CoV-2 and list the negative effects of the infection:

*o	(page 2-line 26): “During the severe acute respiratory syndrome coronavirus 2 (SARS-CoV-2) pandemic, communication between…..”*

*o	(page 4-line 74): “While some studies showed an increased risk of complications in infected pregnant women(such as admission to an intensive care unit, invasive ventilation, need for extra corporeal membrane oxygenation, preterm delivery or admission to a neonatal unit for their newborn)[3–5] and a greater possibility of vertical transmission of the virus [6], others found no increased risk compared with non-pregnant women [7–9] and no possible transmission of SARS-CoV-2 from mother to foetus [7,9–12]”*

2) Explain the measured variables.

3) The significance of the study needs more details. What will the study add to the knowledge?

**Answers to questions 2 and 3 :**
Thank you very much for your suggestions to improve the manuscript. We have explained what we mean by “need to communicate” in several places in the text and clarified the contribution of this study.

*o	(page 4-line 79): “These contradictory findings may have generated an increased need for pregnant women to communicate with health professionals about the risks linked to infection (regarding themselves, their foetus or newborn), but also about the course of their pregnancy monitoring or childbirth.”*

*o	(page 5-line 109): “In the literature, pregnant women’s need to communicate with a health professional during the SARS-CoV-2 pandemic about the risks associated with their own infection or that of their foetus/newborn, about the course of pregnancy monitoring or about childbirth, is poorly documented. Yet descriptive information on this subject is necessary, particularly to characterise the women most at risk of having unmet communication needs, in order to develop targeted strategies to improve these women’s quality of life during pregnancy, a period of great vulnerability in terms of mental health.”*

4) Add the study hypothesis.

**Answer 4:**

Our hypothesis was that pregnant women may not have been able to communicate as they wished with health professionals during the lockdown. We wanted to know to what extent this was the case and which women had unmet communication needs. We explain this in the following sentence:

*o	(page 5-line 104):”* *Despite these changes, the overall unprecedented context may have had an impact on the ability of health professionals to respond to their patients’ questions.”*

**Methods:**

1) The study design, ethics, and setting are not clear.

2) How and who administrates the data collection?

3) Who enrolled participants?

4) Who assigned participants?

5) Mention the settings and locations where the data were collected.

**Answers to these 5 questions:**

Thank you for these comments that underline the need for clarification. Covimater is a cross-sectional internet study which took place in July 2020, i.e., two months after the French lockdown (March to May 2020). The women who took part in Covimater had previously given their agreement to participate in a panel of Internet-using parents or future parents. This panel is managed for Santé publique France by a service provider: BVA group. BVA is a company specialized in highly customizable surveys that target specific populations. Santé publique France can then question these panels of internet users on various subjects through BVA (which has exclusive access to the internet users' email addresses) as part of a contractual service. The people contacted by BVA by e-mail were informed of the purpose of the study and chose freely to participate in Covimater. The sample was then constituted using the quota method in order to obtain a structure similar to the population of pregnant women in France. After enrolling the participants (women residenting in metropolitan France), BVA administrated the questionnaires and collected the data. Only pseudonymised databases were then transmitted to Santé Publique France. The data are stored on Santé publique France's servers, respecting the agency’s standards of data security and confidentiality.

We clarified the text at various points in the sections “study design, setting and sample size of Covimater”, “Ethics and endpoint” as follows :

*o	(page 6- study design, setting and sample size of Covimater - lines 127-156):”* *At our request, a service provider (BVA group) interviewed its unpaid pre-pandemic internet panel of 15,000 future parents or parents of children under 3 years of age in order to create a pseudonymised non-probability sample of 500 pregnant adult women who met the inclusion criteria (described below) and volunteered to participate our survey. Covimater is a cross-sectional study using quota sampling, whereby the study sample was assigned a structure similar to that of the target population (i.e., all pregnant women in France) in order to increase the representativeness. The population of parents of children under one year old -as per the National Institute of Statistics and Economic Studies 2016 census- was used to set the quotas [18]. By its broad representation, the latter was judged as a good proxy for our target population of pregnant women in France. The quotas for mothers of children under one year old were used to calculate weightings using Newton’s algorithm in order to obtain weighted individual data for the statistical analysis presented herein [19]. Specifically, these quotas comprised age group, socio-professional category, region of residence, size of urban area, and parity.*

*Eligible women (see below) for Covimater were invited by BVA to answer a web-based questionnaire between 6-20 July 2020, i.e. two months after the end of the first lockdown in France (March to May 2020). The two-month interval was chosen to avoid the memory bias associated with a longer interval. Demographic/socio-economic data, pandemic and lockdown-related data, participants’ perceptions of the pandemic, data on their pregnancy, their health, and on pregnancy monitoring during first lockdown were collected.*

*We compared our sample to another data source (the National Medical and Administrative Database) in order to validate its representativeness. No significant difference in available data for age group, region of residence or parity was observed between the women participating in Covimater and women in the whole French population who gave birth in a hospital maternity ward in France in 2017 (i.e., 99% of pregnant women in France) [20] . Our study shows, with a power of 99%, a difference of at least 20% concerning the variable of interest (see definition below) between two subgroups of balanced/unbalanced women.”*

*o	(page 7- participants - lines 158-164): “Inclusion criteria: women who were pregnant during the first lockdown (17 March–11 May 2020), aged 18 years and over, and residents in metropolitan France.*

*Exclusion criteria: women who were pregnant during the lockdown but with limited exposure to it: those who delivered in the first two weeks of the lockdown and those whose first week of gestation began during the last two weeks of the lockdown (deducted from the expected date of delivery reported by the women).”*

*o	(page 11- Ethics and endpoint - lines 240-246):* *“Covimater received approval from the Saint Maurice Hospital Ethics Committee on 01/07/2020 (approval number n°2020-1). Internet panel volunteers included in the Covimater study were informed by mail of the study’s purpose then given the choice to participate in the survey. Only pseudonymised databases were transmitted to Santé publique France. The data are stored on Santé publique France's servers, respecting the agency’s data security and confidentiality standards.”*

6) How did you achieve the validity and reliability of the outcome measures?

**Answer 6 :**

At the end of the discussion section, when referring to the strengths and limitations of the study, we mention that the study questionnaire was self-administered and, as such, there is always the risk of recall biases or social desirability bias. Covimater relies on the participants’ answers and we did not have access to medical records to verify those answers.

7) Please, reframe the components (SPICES) for methods
i. Study design, setting, sample size
ii. Participant (inclusion and exclusion criteria"
iii. Issue of interest (exposure)
iv. Comparisons
v. Ethics and endpoint
vi. Statistical analysis

We have restructured the "methods" section as recommended.

7) How was the sample size determined?

**Answer 7:**

Santé publique France estimated that the recruitment of a minimum of 500 adult women would allow to anticipate a maximum theoretical margin of error of +/- 4.3% with a confidence interval of 95%. Moreover, knowing the targeting of the Internet panel (panel of parents or future parents using the Internet), the service provider BVA had indicated to us that it would be difficult to recruit a larger number of pregnant women in the desired time frame (two weeks) during the lockdown.

**Discussion:**

1) The strengths should be demonstrated in detail.

**Answer 1:**

Thank you for this suggestion. We highlight Covimater's third strengths as follows :

*o	(page 24-lines 528-539):”In the literature, little information was available on patients’ needs for communication with health professionals, even less in the context of the ongoing SARS-CoV-2 pandemic. Covimater brought new insight to this important topic and identifies specific groups of women who should be targeted by public policies, which constitutes the study’s primary strength. Secondly, Covimater included women with different gestational ages, unlike studies from other countries that mostly focus on the third trimester of pregnancy during the current pandemic. Furthermore, Covimater succeeded in identifying significant associations with the variable of interest despite the fact that some of the groups compared were unbalanced in size (with consequently reduced power).”*

2) Add your recommendations with study implications.

**Answer 2:**

As recommended, we add a sentence summarising our recommendations:

*o	(page 24-lines 522-527): “Our work therefore highlighted the importance of maintaining or promoting communication between health professionals and pregnant women during the pandemic. Specific information campaigns could also be enhanced and circulated through diverse media channels (e.g., radio or television) to help reassure this population during the pandemic.”*

3) The main limitation of the study is not demonstrated.

**Answer 3:**

Thank you for this suggestion. It’s very difficult to demonstrate the inclusion bias inherent to quota sampling studies or the fact that pregnant women accept to participate to this study or the panel of parents. We therefore prefer to alert readers so they remain aware of this weakness when considering results from the Covimater study. We have tried to clarify as follows:

*o	(page 25-lines 541-552): “Covimater also had some limitations. First, the use of a panel of volunteers and quota sampling could imply an inclusion bias in the pregnant women who accepted to participate for the survey. However, no alternative method available would have permitted the study to take place in sufficient time to avoid a significant recall bias. The further away the lockdown was the more difficult it would have been to collect reliable information from women about their behaviour and feelings during the period. Consequently, greater caution is required when interpreting the statistical inference of our results than would be needed for random sample studies.*”
